# Results From the First Six Years of National Sentinel Surveillance for Influenza in Kenya, July 2007–June 2013

**DOI:** 10.1371/journal.pone.0098615

**Published:** 2014-06-23

**Authors:** Mark A. Katz, Philip Muthoka, Gideon O. Emukule, Rosalia Kalani, Henry Njuguna, Lilian W. Waiboci, Jamal A. Ahmed, Godfrey Bigogo, Daniel R. Feikin, Moses K. Njenga, Robert F. Breiman, Joshua A. Mott

**Affiliations:** 1 Centers for Disease Control and Prevention-Kenya/Kenya Medical Research Institute, Nairobi, Kenya; 2 Kenya Ministry of Health, Nairobi, Kenya; Naval Research Laboratory, United States of America

## Abstract

**Background:**

Recent studies have shown that influenza is associated with significant disease burden in many countries in the tropics, but until recently national surveillance for influenza was not conducted in most countries in Africa.

**Methods:**

In 2007, the Kenyan Ministry of Health with technical support from the CDC-Kenya established a national sentinel surveillance system for influenza. At 11 hospitals, for every hospitalized patient with severe acute respiratory illness (SARI), and for the first three outpatients with influenza-like illness (ILI) per day, we collected both nasopharyngeal and oropharyngeal swabs. Beginning in 2008, we conducted in-hospital follow-up for SARI patients to determine outcome. Specimens were tested by real time RT-PCR for influenza A and B. Influenza A-positive specimens were subtyped for H1, H3, H5, and (beginning in May 2009) A(H1N1)pdm09.

**Results:**

From July 1, 2007 through June 30, 2013, we collected specimens from 24,762 SARI and 14,013 ILI patients. For SARI and ILI case-patients, the median ages were 12 months and 16 months, respectively, and 44% and 47% were female. In all, 2,378 (9.6%) SARI cases and 2,041 (14.6%) ILI cases were positive for influenza viruses. Most influenza-associated SARI cases (58.6%) were in children <2 years old. Of all influenza-positive specimens, 78% were influenza A, 21% were influenza B, and 1% were influenza A/B coinfections. Influenza circulated in every month. In four of the six years influenza activity peaked during July–November. Of 9,419 SARI patients, 2.7% died; the median length of hospitalization was 4 days.

**Conclusions:**

During six years of surveillance in Kenya, influenza was associated with nearly 10 percent of hospitalized SARI cases and one-sixth of outpatient ILI cases. Most influenza-associated SARI and ILI cases were in children <2 years old; interventions to reduce the burden of influenza, such as vaccine, could consider young children as a priority group.

## Introduction

Influenza causes substantial morbidity and mortality worldwide every year. In 2008, in children <5 years old, an estimated 90 million new cases of influenza occurred, including an estimated 28,000–111,500 deaths, nearly all of which (99%) occurred in developing countries [1]. While seasonal influenza has long been recognized as a cause of morbidity and mortality in developed countries with temperate climates, recent studies have shown that influenza causes a significant burden of disease in countries throughout the tropics as well [2]–[4].

In Sub-Saharan Africa, surveillance for influenza has been extremely limited, likely due to a combination of limited public health infrastructure in the region, and competing health priorities such as HIV, tuberculosis, and malaria [5]–[9]. However, in 2005, in response to the threat of avian influenza A(H5N1), governments throughout the world and international organizations such as the World Health Organization (WHO) began investing more resources in influenza pandemic preparedness. As a result, a number of countries in Africa were able to use public health resources to improve their capacity to conduct virological and epidemiological surveillance for influenza [10], [11].

In Kenya, an equatorial country in East Africa with a mostly tropical climate, few data are available on influenza. In 2006, the Kenyan Ministry of Health, with technical support from the Centers for Disease Control and Prevention-Kenya (CDC-K), established a national sentinel surveillance system for influenza. The objectives of the surveillance system were to identify circulating influenza strains, to understand the epidemiology and burden of influenza in Kenya, and to serve as a component of an early warning system for pandemic influenza.

## Methods

### Study sites

**Figure 1:**
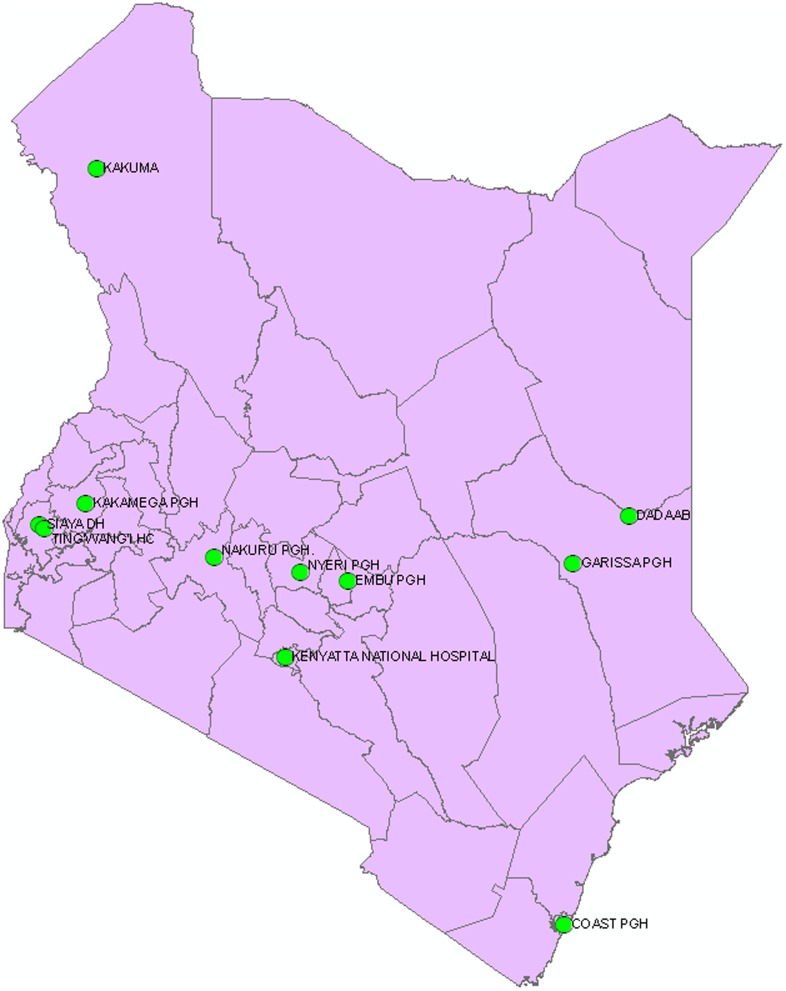
Map of Kenya showing the distribution of influenza sentinel surveillance site.

Beginning in August 2006, The Kenyan Ministry of Health (MOH) and CDC-K initiated influenza surveillance at a national referral hospital (Kenyatta National Hospital) in Nairobi, seven provincial general hospitals (Coast, Embu, Garissa, Kakamega, Nakuru, New Nyanza and Nyeri), and two refugee camps (Kakuma and Dadaab) (Figure 1). At each site, surveillance was conducted for influenza-like illness (ILI) and severe acute respiratory illness (SARI). In August 2009, in order to integrate influenza surveillance with other ongoing surveillance activities in Nyanza Province, we initiated SARI surveillance at Siaya District Hospital (SDH) and ILI surveillance at Tingwang'i Health Center (THC), and discontinued influenza surveillance at New Nyanza Provincial Hospital.

A trained surveillance officer – either a nurse or a clinical officer – was employed at each of the sites. Officers identified ILI patients at outpatient clinics and emergency rooms, and SARI patients in adult and pediatric in-patient wards in the hospitals. From August 2006 through December 2011, for every consented person with SARI and for the first three consented ILI patients per weekday at each site, a two-page questionnaire with questions about demographics, underlying diseases, influenza vaccine history, signs and symptoms, and exposures, was administered, and a combined nasopharyngeal (NP) swab and oropharyngeal (OP) swab was collected. From January 2012–June 2013, surveillance was conducted at nine hospitals only (surveillance at Embu and Garissa was discontinued) and was limited to SARI in all but 2 locations (Kenyatta National Hospital and Tingwan'I Health Center). Beginning in 2008, we conducted in-hospital follow-up for SARI patients to determine final outcome (discharge or death). Deaths that occurred within 30 days of hospital admission were considered to be associated with SARI.

### Case Definitions

The following case definitions were used:

#### Influenza-Like Illness

Influenza-like illness was defined as an axillary temperature ≥38°C and cough or sore throat in an outpatient of any age.

#### Severe Acute Respiratory Illness

The case definition used for severe acute respiratory illness was different for persons <5 and those ≥5 years old. For children <5 years old, a modified version of the World Health Organization's Integrated Management of Childhood Illness (IMCI) definition for pneumonia [12] was used, requiring the presence of cough or difficulty breathing plus one of the following danger signs: chest in-drawing, stridor, unable to breastfeed or drink, vomits everything, convulsions, lethargy, or unconsciousness. For patients ≥5 years old, SARI was defined as axillary temperature ≥38°C plus cough, difficulty breathing or shortness of breath. Hospitalization was a required criterion for SARI cases, regardless of age.

### Specimen collection

For each patient, NP and OP swabs were placed into a single cryovial with viral transport medium (VTM), which was prepared at the Kenya Medical Research Institute(KEMRI)/CDC laboratory in Nairobi according to a WHO protocol [13] and routinely distributed to each of the surveillance sites. The procedure for specimen collection has been previously described [14]. Specimens were immediately refrigerated at 2–8 degrees Celsius. Specimens from all sites underwent triple packaging, and were transported by courier service or hand-carried to the National Influenza Center (NIC) in Nairobi, where they were immediately placed in a −80°Celsius freezer. Samples from the two refugee camps were transported directly to the KEMRI/CDC-K laboratory in Nairobi by scheduled flights.

### Laboratory Testing

At the NIC and the KEMRI/CDC laboratories, specimens were tested by real time reverse transcription-polymerase chain reaction (rRT-PCR) for influenza A and influenza B viruses. Total *ribonucleic acid* (RNA) was extracted from 140 µL aliquots of each specimen using a QIAamp viral RNA mini kit (Qiagen GmbH, Germany) according to the manufacturer's instructions. One step rRT-PCR was carried out using the AgPath kit (Applied Biosystems, California, USA). Following the reverse transcription step, a typical 45 cycle PCR reaction was run and fluorescence was read at the annealing/extension step. Appropriate negative and positive control specimens were run alongside each reaction. The results were recorded as cyclic threshold (*C_T_*) values. When all controls met the stated requirements, any influenza A and B *C_T_* value <40 was recorded as positive. Values with a *C_T_* reading ≥40 were recorded as negative.

All specimens positive for influenza A were sub-typed for H1, H3, H5, and (beginning in May 2009) pH1N1 using rRT-PCR. Specimens that were positive for influenza A virus by rRT-PCR but failed to sub-type were sent to the WHO Influenza Collaborating Center at CDC-Atlanta, GA, USA for further antigenic characterization.

### Data Management

Data from all but two sites were collected using standard paper questionnaires and entered into a Microsoft Access database. At Siaya District Hospital and Tingwang'i Health Center, scannable paper forms were used (TeleForm software, Cardiff, Vista, CA). Beginning in May 2011 smart phones (HTC Touch Pro 2 model) were used to collect the same demographic and clinical data from patients in five hospitals. Data collected on smart phones were uploaded daily to the KEMRI/CDC server in Kisumu using a secure web link. Data were downloaded from the server onto an MS Access database. We conducted data cleaning to check for inconsistent or illogical data every week. Laboratory data were merged with epidemiologic data weekly.

### Data Analysis

Data from July 2007, the first month when surveillance was conducted in all 11 sites, through June 2013, were included in the analysis. We excluded patients for whom demographic data or laboratory results were missing, and patients who did not meet one of the case definitions. We used Pearson's chi square test to compare demographic and clinical variables between SARI and ILI patients. We used logistic regression to calculate odds ratios in bi-variate and multivariate analyses comparing influenza-positive and influenza-negative ILI patients, and influenza-positive and influenza-negative SARI patients. We used generalized linear models (GLM) to estimate relative risk when comparing fatal and non-fatal cases. Multivariable logistic regression and GLM models were constructed using factors that were significant at p<0.2 in the bi-variate analysis. SARI patients <5 years old and those ≥5 years old were analyzed separately because different case definitions were used for the two age groups. Findings were considered statistically significant if the p-value was <0.05. Data analyses were performed using Stata 12.1 (Stata Corp. 2011. Stata Statistical Software: Release 12. College Station, TX: Stata Corp LP).

### Ethical considerations

The Kenya Ministry of Health (KMoH) issued a document stating that sentinel surveillance for influenza, including follow-up in-hospital surveillance, should be considered part of routine public health surveillance, and therefore did not require formal ethical review. Because the activity was classified as routine surveillance, the KMoH considered verbal consent to be adequate. Verbal consent was obtained from all patients before questionnaires were administered and specimens were collected. For children, verbal consent was obtained from guardians. Surveillance officers documented consenting and non-consenting participants in a log book at each site. Authors did not participate in sample collection. Data was anonymized upon collection, and authors did not have access to identifying information.

## Results

From July 1, 2007 through June 30, 2013, a total of 38,775 (24,762 SARI and 14,013 ILI) specimens were collected from patients in the 11 sentinel surveillance sites. Overall, 90% of SARI samples and 87% of ILI samples were collected from children <5 years (Table 1). The median ages of SARI and ILI cases were 1 year (range: 1 month–95 years) and 1 year and 4 months (range: 1 month–75 years), respectively. Of the SARI and ILI cases, 10,823 (44%) and 6,582 (47%) were female, respectively. A significantly greater percentage of SARI patients reported having any underlying and chronic conditions compared to ILI patients (6.8% vs. 2.3%, p<0.001). The most common underlying medical conditions and chronic symptoms reported were recurrent chest pain (2.0%) and asthma (1.5%) among SARI patients, and asthma (0.6%) and chronic shortness of breath (0.5%) among ILI patients. The median duration of illness from the date of symptom onset to presentation at the health facility was three days [interquartile range (IQR): 2–5 days] for SARI patients, and 2 days (IQR: 1–3 days) for ILI patients. Although overall 1.5% of all SARI and ILI patients reported having received influenza vaccination in the previous year, the majority of these patients (60%) were from Kakuma Refugee Camp (data not shown), where free seasonal influenza vaccine was distributed by the Kenyan Ministry of Public Health and Sanitation in 2010. Overall 16.0% of SARI patients <5 years old, 18.6% of SARI patients ≥5 years old and 9.7% of all ILI patients had been hospitalized during the previous year.

**Table 1:** Characteristics of patients and comparison of SARI and ILI, Kenya, July 2007–June 2013.

	SARI (N = 24,762)	ILI (N = 14,013)	
	n (%)	n (%)	p-value
**Sex**			
Female	10823(43.7)	6582(47.0)	<0.001
**Age Group**			
0–23 months	16709(67.5)	7916(56.5)	<0.001
24–59 months	5632(22.7)	4281(30.6)	
5–9 years	1247(5.0)	1199(8.6)	
10–17 years	360(1.5)	269(1.9)	
18–49 years	684(2.8)	321(2.3)	
≥50 years	130(0.5)	27(0.2)	
Mean age years (SD)	2.8(6.9)	2.9(5.2)	
Median age years (IQR)	1.0(0.5–2.0)	1.3(0.8–3.0)	
**Site**			
Kenyatta NH	2138(8.6)	1443(10.3)	<0.001
Coast PGH	2112(8.5)	809(5.8)	
Nakuru PGH	2094(8.5)	1869(13.3)	
Nyeri PGH	3118(12.6)	1999(14.3)	
Kakamega PGH	4300(17.4)	2022(14.4)	
Embu PGH	430(1.7)	794(5.7)	
Garissa PGH	764(3.1)	546(3.9)	
Kakuma	3705(15.0)	1477(10.5)	
Dadaab	2880(11.6)	1303(9.3)	
Siaya DH	3125(12.6)	0(0.0)	
Ting'wang'i HC	0(0.0)	1649(11.8)	
Nyanza PGH	96(0.4)	102(0.7)	
**Length of illness before presenting to the hospital**			
0–3 days	14299(57.7)	10588(75.6)	<0.001
4–7 days	8959(36.2)	3037(21.7)	
>7 days	1504(6.1)	388(2.8)	
Mean duration of illness (SD)	3.7(2.8)	2.6(2.5)	
Median duartion of illness (IQR)	3.0(2.0–5.0)	2.0(1.0–3.0)	
**Underlying chronic illnesses** a			
Any chronic illness	1695(6.8)	327(2.3)	<0.001
Heart disease	114(0.5)	25(0.2)	<0.001
Chronic shortness of breath	269(1.1)	70(0.5)	<0.001
Recurrent chest pain	507(2.0)	16(0.1)	<0.001
Asthma	370(1.5)	85(0.6)	<0.001
Chronic cough (≥3 months in 2 consecutive years)	144(0.6)	58(0.4)	0.021
Active TB	147(0.6)	19(0.1)	<0.001
**Reported vaccination against influenza in the past year**	375(1.5)	222(1.6)	0.943
**Influenza Viruses**			
Influenza A and/or B	2378(9.6)	2041(14.6)	<0.001
*2007*	250/2,245(11.1)	238/1,505(15.8)	
*2008*	354/4,106(8.6)	378/2,883(13.1)	
*2009*	504/4,200(12.0)	609/3,351(18.2)	
*2010*	458/4,775(9.6)	421/3,361(12.5)	
*2011*	503/5,593(9.0)	343/2,224(15.4)	
*2012*	199/2,758(7.2)	39/479(8.1)	
*2013*	110/1,085(10.1)	13/210(6.2)	
Influenza A	1853(7.5)	1621(11.6)	<0.001
*Pandemic H1N1* *	421/1,313 (32.1)	391/1,054(37.1)	
*Seasonal A (H1N1)* *	138/1,853(7.4)	218/1,621(13.4)	
*Seasonal A (H3N2)* *	478/1,853(25.8)	407/1,621(25.1)	
*Unsubtyped* * b	0/1,853(0.0)	4/1,621(0.2)	
*Not subtyped* * c	817/1,853(44.1)	603/1,621(37.2)	
Influenza B	545(2.2)	462(3.3)	<0.001
Influenza A and B co-infection	20(0.1)	42(0.3)	<0.001

a These data were not collected in Siaya DH and Tingwangi.

b Flu A positive samples with CT values ≤37 which could not be subtyped.

c Flu A positive samples that were not subtyped. Most of these (95%) were samples tested before June 2008 when subtyping started.

*Denominator = Total Influenza A Cases.

In all, 2,378 (9.6% of total) SARI cases and 2,041 (14.6%) of ILI cases were positive for influenza viruses (p<0.001, Table 1). For years in which a complete calendar year of surveillance was conducted, the percentage of influenza-positive SARI cases ranged from a low of 7.2% in 2012 to a high of 12.0% in 2009, while the percentage of influenza-positive ILI cases ranged from a low of 8.1% in 2012 to a high of 18.2% in 2009. Of all influenza-positive SARI specimens, 77.1% were influenza A, 22.1% were influenza B, and 0.8% were co-infections with influenza A and B. Among the influenza-positive ILI samples collected, 77.2% were influenza A, 20.7% were influenza B, and 2.1% were co-infections. Most influenza A samples that were subtyped were either H3 (25.8% and 25.1% of SARI and ILI patients, respectively) or A(H1N1) pdm09 (32.1% and 37.1% of SARI and ILI patients tested after May 2009, respectively).

**Figure 2:**
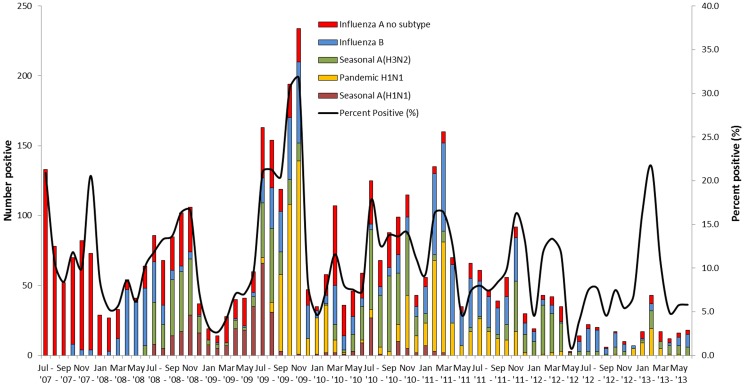
Seasonality of influenza for ILI and SARI patients, Kenya, July 2007–June 2013. Note: Smoothing done using the double exponential smoothing methods.

In the six years of surveillance, influenza circulated nearly every month. There were periods of increased influenza activity, particularly during the initial months that pandemic influenza A (H1N1) virus circulated in Kenya in late 2009. In four of the six years (2008–2011) the highest absolute number of influenza-positive samples and the highest percentage-positive of SARI specimens for influenza occurred during July to November (Figure 2). Influenza B and influenza A (H3N2) viruses circulated throughout the six years. Influenza A (H1N1)pdm 09 virus circulated consistently after its first observation in July 2009, while seasonal H1N1 virus was prominent in 2008 and 2009, and appeared sporadically in 2010.

Among SARI patients <5 years old, there was a higher percentage of influenza positivity in children 24–59 months old compared to patients ≤23 months old [adjusted Odds Ratio (aOR) = 1.3, 95% Confidence Interval (CI) = 1.2–1.5] (Table 2). Asthma was less common in influenza-positive children compared to influenza-negative children (aOR = 0.5, 95% CI = 0.3–0.9). Fever and convulsions were slightly more common in influenza-positive patients, while weight loss was more common in influenza-negative children. Among SARI patients ≥5 years old, no demographic factors were associated with influenza cases, and headache was the only symptom more common in influenza-positive cases compared with influenza-negative cases (aOR = 1.4 (95% CI = 1.1–2.0) (Table 3). For ILI and SARI patients <5 years old and ≥5 years old, in multivariate analysis, the percentage of influenza positivity varied by hospital (p<0.001 for all three comparisons).

**Table 2:** Laboratory-confirmed influenza infection among SARI patients aged <5 years, by demographic and clinical characteristics, at 9 sites in Kenya, July 2007–June 2013.

	Total (N = 22,341)	Influenza positive patients (N = 2,021)	Influenza negative patients (N = 20,320)	Crude OR (95% CI)	p-Value	Adjusted^a^ OR (95% CI)	p-Value
		n(%)¥	n(%)¥				
**Sex**							
Male	12,644	1170(9.3)	11474(90.7)	Ref			
Female	9,697	851(8.8)	8846(91.2)	0.9(0.9–1.0)	0.218	-	-
**Age Group**							
0–23 months	16,709	1395(8.3)	15314(91.7)	Ref		Ref	
24–59 months	5,632	626(11.1)	5006(88.9)	1.4(1.2–1.5)	<0.001	1.3(1.2–1.5)	<0.001
**Site**							
Kenyatta NH	2,084	137(6.6)	1947(93.4)	121.6^b^	<0.001	-	-
Coast PGH	2,020	164(8.1)	1856(91.9)				
Nakuru PGH	1,954	214(11.0)	1740(89.0)				
Nyeri PGH	2,890	325(11.2)	2565(88.8)				
Kakamega PGH	3,655	289(7.9)	3366(92.1)				
Embu PGH	405	19(4.7)	386(95.3)				
Garissa PGH	733	65(8.9)	668(91.1)				
Kakuma	3,206	336(10.5)	2870(89.5)				
Dadaab	2,362	284(12.0)	2078(88.0)				
Siaya DH	3,032	188(6.2)	2844(93.8)				
Nyanza PGH							
**Length of illness before presenting to the hospital**							
0–3 days	12,996	1183(9.1)	11813(90.9)	Ref		Ref	
4–7 days	8,031	707(8.8)	7324(91.2)	1.0(0.9–1.1)	0.461	1.1(0.9–1.2)	0.259
>7 days	1,314	131(10.0)	1183(90.0)	1.1(0.9–1.3)	0.300	1.3(1.0–1.7)	0.040
Have children or take care of children in the household (Yes vs. No)	10,900	1024(9.4)	9876(90.6)	1.1(1.0–1.2)	0.122	1.1(1.0–1.2)	0.184
**Underlying chronic disease** c							
Any chronic disease (Yes vs. No)	1,371	125(9.1)	1246(90.9)	1.0(0.8–1.2)	0.644	-	-
Heart disease (Yes vs. No)	95	4(4.2)	91(95.8)	0.4(0.2–1.2)	0.096	0.7(0.3–2.1)	0.571
Chronic shortness of breath (Yes vs. No)	237	17(7.2)	220(92.8)	0.7(0.5–1.2)	0.237	-	
Recurrent chest pain (Yes vs. No)	458	71(15.5)	387(84.5)	1.8(1.4–2.3)	<0.001	1.3(1.0–1.8)	0.085
Asthma (Yes vs. No)	309	14(4.5)	295(95.5)	0.5(0.3–0.8)	0.004	0.5(0.3–0.9)	0.023
Chronic cough (Yes vs. No)	102	16(15.7)	86(84.3)	1.8(1.1–3.1)	0.032	1.7(0.9–3.1)	0.084
Active Tb (Yes vs. No)	53	1(1.9)	52(98.1)	0.2(0.0–1.4)	0.096	0.3(0.0–2.0)	0.202
Hospitalized in the last 12 months (Yes vs. No)	3,576	341(9.5)	3235(90.5)	1.1(0.9–1.2)	0.356	-	
Had contact with anyone with a similar illness in the last 3 week (Yes vs. No)	1,839	168(9.1)	1671(90.9)	1.0(0.8–1.2)	0.765	-	
Vaccinated against influenza in the past year (Yes vs. No)	295	22(7.5)	273(92.5)	0.9(0.6–1.4)	0.576	-	
**Signs and symptoms within the last 14 days** *							
Reported or Documented Fever (Yes vs. No)	18,408	1770(9.6)	16638(90.4)	1.6(1.4–1.8)	<0.001	1.4(1.2–1.8)	<0.001
Weight loss (Yes vs. No)	5,377	399(7.4)	4978(92.6)	0.8(0.7–0.8)	<0.001	0.8(0.7–0.9)	0.002
Convulsions (Yes vs. No)	5,242	528(10.1)	4714(89.9)	1.2(1.0–1.3)	0.005	1.2(1.0–1.3)	0.032
Stridor (Yes vs. No)	4,042	400(9.9)	3642(90.1)	1.1(1.0–1.3)	0.027	1.0(0.9–1.2)	0.899
Nasal flaring (Yes vs. No)	9,482	880(9.3)	8602(90.7)	1.0(0.9–1.1)	0.369	-	
Tachypnea (Yes vs. No)^c^	6,278	561(8.9)	5717(91.1)	0.9(0.8–1.0)	0.194	0.9(0.8–1.1)	0.215
Chest in-drawing	8,234	726(8.8)	7508(91.2)	1.0(0.9–1.1)	0.368	-	
Grunting (Yes vs. No)^c^	7,353	705(9.6)	6648(90.4)	1.1(1.0–1.2)	0.316	-	
Unable to drink or breastfeed at all	7,234	678(9.4)	6556(90.6)	1.1(1.0–1.2)	0.143	1.0(0.9–1.2)	0.966
Lethargic (Yes vs. No)	6,370	624(9.8)	5746(90.2)	1.1(1.0–1.3)	0.014	1.1(0.9–1.3)	0.263
Loss of consciousness (Yes vs. No)	1,853	218(11.8)	1635(88.2)	1.4(1.2–1.6)	<0.001	1.0(0.8–1.3)	0.804
Headache (Yes vs. No)	769	103(13.4)	666(86.6)	1.6(1.3–1.9)	<0.001	1.2(0.9–1.5)	0.146
Sore throat (Yes vs. No)	1,977	197(10.0)	1780(90.0)	1.1(1.0–1.3)	0.136	1.0(0.8–1.2)	0.958
Nausea (Yes vs. No)	4,040	419(10.4)	3621(89.6)	1.2(1.1–1.3)	0.002	1.0(0.9–1.2)	0.658
Vomiting (Yes vs. No)	12,270	1131(9.2)	11139(90.8)	1.0(1.0–1.1)	0.324	-	

a Adjusted for variables that were significant at p<.2 in the bivariate analysis and data collection site;

b Pearson chi-square;

c Data not collected in Siaya DH and Tingwangi;

¥ Row percentages shown.

*Signs such as stridor, nasal flaring, tachypnea, chest in-drawing and grunting were evaluated at the time of patient presentation to the hospital.

**Table 3:** Odds of testing positive for influenza for various demographic and clinical characteristics among SARI patients aged ≥5 years in Kenya, July 2007–June 2013.

	Total (N = 2,421)	Influenza positive patients (N = 357)	Influenza negative patients (N = 2,064)	Crude OR (95% CI)	p-Value	Adjusted^a^ OR (95% CI)	p-Value
		n(%)¥	n(%)¥				
**Sex**							
Male	1,295	187(14.4)	1108(85.6)	Ref			
Female	1,126	170(15.1)	956(84.9)	1.1(0.8–1.3)	0.649	-	
**Age Group**							
5–9 years	1,247	204(16.4)	1043(83.6)	Ref		Ref	
10–17 years	360	58(16.1)	302(83.9)	1.0(0.7–1.4)	0.911	0.9(0.6–1.3)	0.484
18–49 years	684	81(11.8)	603(88.2)	0.7(0.5–0.9)	0.008	0.7(0.4–1.1)	0.086
≥50 years	130	14(10.8)	116(89.2)	0.6(0.3–1.1)	0.100	0.8(0.4–1.8)	0.627
**Site**							
Kenyatta NH	54	1(1.9)	53(98.1)	Ref			
Coast PGH	92	7(7.6)	85(92.4)	77.8^b^	<0.001^c^	-	-
Nakuru PGH	140	16(11.4)	124(88.6)				
Nyeri PGH	228	34(14.9)	194(85.1)				
Kakamega PGH	645	73(11.3)	572(88.7)				
Embu PGH	25	0(0.0)	25(100.0)				
Garissa PGH	31	9(29.0)	22(71.0)				
Kakuma	499	67(13.4)	432(86.6)				
Dadaab	518	130(25.1)	388(74.9)				
Siaya DH	93	14(15.1)	79(84.9)				
Nyanza PGH	96	6(6.3)	90(93.8)				
**Length of illness before presenting to the hospital**							
0–3 days	1,303	215(16.5)	1088(83.5)	Ref			
4–7 days	928	125(13.5)	803(86.5)	0.8(0.6–1.0)	0.050	1.1(0.8–1.5)	0.568
>7 days	190	17(8.9)	173(91.1)	0.5(0.3–0.8)	0.008	0.7(0.4–1.5)	0.396
Have children or take care of children in the household (Yes vs. No)	1,323	169(12.8)	1154(87.2)	1.2(0.9–1.7)	0.166	1.4(0.9–2.0)	0.094
**Underlying chronic disease** d							
Any chronic disease (Yes vs. No)	324	31(9.6)	293(90.4)	0.6(0.4–0.9)	0.005	0.8(0.5–1.4)	0.453
Heart disease (Yes vs. No)	19	1(5.3)	18(94.7)	0.3(0.0–2.3)	0.249		
Chronic shortness of breath (Yes vs. No)	32	3(9.4)	29(90.6)	0.6(0.2–1.9)	0.347		
Recurrent chest pain (Yes vs. No)	49	9(18.4)	40(81.6)	1.2(0.6–2.6)	0.559		
Asthma (Yes vs. No)	61	5(8.2)	56(91.8)	0.5(0.2–1.2)	0.127	0.8(0.3–2.7)	0.754
Chronic cough (Yes vs. No)	42	5(11.9)	37(88.1)	0.7(0.3–1.9)	0.528		
Active Tb (Yes vs. No)	94	6(6.4)	88(93.6)	0.4(0.2–0.9)	0.020	1.0(0.3–3.1)	0.999
Hospitalized in the last 12 months (Yes vs. No)	450	57(12.7)	393(87.3)	1.1(0.8–1.5)	0.612		
Had contact with anyone with a similar illness in the last 3 week (Yes vs. No)	46	9(19.6)	37(80.4)	1.6(0.8–3.3)	0.227		
Currently smoking (Yes vs. No)	112	11(9.8)	101(90.2)	0.5(0.3–1.0)	0.056	1.1(0.5–2.5)	0.745
Vaccinated against influenza in the past year (Yes vs. No)	80	7(8.8)	73(91.3)	0.7(0.3–1.7)	0.469		
**Symptoms within the last 14 days**							
Difficulty in breathing/shortness of breath (Yes vs. No)	1,850	278(15.0)	1572(85.0)	1.1(0.8–1.4)	0.483		
Sore throat (Yes vs. No)	805	113(14.0)	692(86.0)	0.9(0.7–1.2)	0.488		
Weight loss (Yes vs. No)	766	91(11.9)	675(88.1)	0.7(0.5–0.9)	0.006	0.8(0.5–1.1)	0.214
Convulsions (Yes vs. No)	387	62(16.0)	325(84.0)	1.1(0.8–1.5)	0.463		
Lethargic (Yes vs. No)	493	100(20.3)	393(79.7)	1.7(1.3–2.1)	<0.001	1.1(0.8–1.7)	0.505
Loss of consciousness (Yes vs. No)	257	58(22.6)	199(77.4)	1.8(1.3–2.5)	<0.001	1.0(0.6–1.5)	0.868
Headache (Yes vs. No)	1,086	189(17.4)	897(82.6)	1.5(1.2–1.8)	0.001	1.5(1.1–2.0)	0.018
Nausea (Yes vs. No)	631	113(17.9)	518(82.1)	1.4(1.1–1.8)	0.011	1.0(0.7–1.4)	0.965
Vomiting (Yes vs. No)	1,187	178(15.0)	1009(85.0)	1.0(0.8–1.3)	0.734		
Muscle pains (Yes vs. No)	536	81(15.1)	455(84.9)	1.0(0.8–1.4)	0.821	-	

a Adjusted for variables that were significant at p<.2 in the bivariate analysis and data collection site;

b Pearson chi-square;

c Fisher's exact p-value;

d Data not collected in Siaya DH and Tingwangi;

¥ Row percentages shown.

Among ILI cases, children ≤23 months old had the lowest percentage of influenza-positive specimens [844/7916 (10.7%] compared to all other age groups. ILI patients who presented to the hospital after 7 days of illness were less likely to be influenza-positive compared to ILI patients with shorter presentations. There were no other demographic characteristics or clinical signs or symptoms that were significantly associated with either influenza-positive or influenza-negative ILI patients (Table 4).

**Table 4:** Odds of testing positive for influenza for various demographic and clinical characteristics among ILI patients in Kenya, Jul 2007–Jun 2013.

	Total (N = 14,013)	Influenza positive patients (N = 2,041)	Influenza negative patients (N = 11,972)	Crude OR (95% CI)	p-Value	Adjusted^a^ OR (95% CI)	p-Value
		n(%)¥	n(%)¥				
**Sex**							
Male	7,431	1069(14.4)	6362(85.6)	Ref			
Female	6,582	972(14.8)	5610(85.2)	1.0(0.9–1.1)	0.522	**-**	
**Age Group**							
0–23 months	7,916	844(10.7)	7072(89.3)	Ref		Ref	
24–59 months	4,281	761(17.8)	3520(82.2)	1.8(1.6–2.0)	<0.001	1.8(1.6–2.0)	<0.001
5–9 years	1,199	273(22.8)	926(77.2)	2.5(2.1–2.9)	<0.001	2.5(2.1–2.9)	<0.001
10–17 years	269	81(30.1)	188(69.9)	3.6(2.8–4.7)	<0.001	4.3(3.3–5.8)	<0.001
18–49 years	321	74(23.1)	247(76.9)	2.5(1.9–3.3)	<0.001	3.8(2.8–5.2)	<0.001
≥50 years	27	8(29.6)	19(70.4)	3.5(1.5–8.1)	0.003	5.0(2.1–11.9)	<0.001
**Site**							
Kenyatta NH	1,443	168(11.6)	1275(88.4)	79.5^b^	<0.001	-	-
Coast PGH	809	100(12.4)	709(87.6)				
Nakuru PGH	1,869	315(16.9)	1554(83.1)				
Nyeri PGH	1,999	362(18.1)	1637(81.9)				
Kakamega PGH	2,022	325(16.1)	1697(83.9)				
Embu PGH	794	68(8.6)	726(91.4)				
Garissa PGH	546	91(16.7)	455(83.3)				
Kakuma	1,477	191(12.9)	1286(87.1)				
Dadaab	1,303	200(15.3)	1103(84.7)				
Ting'wang'i HC	1,649	211(12.8)	1438(87.2)				
Nyanza PGH	102	10(9.8)	92(90.2)				
**Length of illness before presenting to the hospital**							
0–3 days	10,588	1572(14.8)	9016(85.2)	Ref		Ref	
4–7 days	3,037	428(14.1)	2609(85.9)	0.9(0.8–1.1)	0.301	1.0(0.8–1.1)	0.520
>7 days	388	41(10.6)	347(89.4)	0.7(0.5–0.9)	0.020	0.7(0.5–0.98)	0.037
Have children or take care of children in the household	6,121	955(15.6)	5166(84.4)	1.2(1.1–1.3)	0.001	1.0(0.9–1.2)	0.529
**Underlying chronic disease** c							
Any chronic disease (Yes vs. No)	327	51(15.6)	276(84.4)	1.1(0.8–1.4)	0.747	-	
Heart disease (Yes vs. No)	25	4(16.0)	21(84.0)	1.1(0.4–3.2)	0.849	-	
Chronic shortness of breath (Yes vs. No)	70	8(11.4)	62(88.6)	0.7(0.4–1.6)	0.437	-	
Recurrent chest pain (Yes vs. No)	16	4(25.0)	12(75.0)	1.9(0.6–6.0)	0.254	-	
Asthma (Yes vs. No)	85	14(16.5)	71(83.5)	1.1(0.6–2.0)	0.635	-	
Chronic cough (Yes vs. No)	58	10(17.2)	48(82.8)	1.2(0.6–2.4)	0.583	-	
Active Tb (Yes vs. No)	19	4(21.1)	15(78.9)	1.6(0.5–4.7)	0.434	-	
Had contact with anyone with a similar illness in the last 3 week (Yes vs. No)	1,489	236(15.8)	1253(84.2)	1.0(0.9–1.2)	0.578	-	
Hospitalized in last 12 months (Yes vs. No)	1,356	195(14.4)	1161(85.6)	0.97(0.8–1.2)	0.750	-	
Vaccinated against influenza in the past year (Yes vs. No)	222	25(11.3)	197(88.7)	0.8(0.5–1.3)	0.356	-	
**Symptoms within the last 14 days**							
Difficulty in breathing/shortness of breath (Yes vs. No)	4,094	577(14.1)	3517(85.9)	0.9(0.9–1.1)	0.310	-	
Weight loss (Yes vs. No)	1,732	237(13.7)	1495(86.3)	0.9(0.8–1.1)	0.250	-	
Convulsions (Yes vs. No)	330	50(15.2)	280(84.8)	1.0(0.8–1.4)	0.773	-	
Lethargic (Yes vs. No)	1,108	135(12.2)	973(87.8)	0.8(0.7–1.0)	0.019	0.8(0.7–1.0)	0.057
Loss of consciousness(Yes vs. No)	137	16(11.7)	121(88.3)	0.8(0.5–1.3)	0.337	-	
Headache (Yes vs. No)	1,636	327(20.0)	1309(80.0)	1.5(1.4–1.8)	<0.001	1.1(1.0–1.3)	0.081
Nausea (Yes vs. No)	1,795	295(16.4)	1500(83.6)	1.2(1.0–1.3)	0.018	1.0(0.9–1.2)	0.621
Vomiting (Yes vs. No)	2,659	369(13.9)	2290(86.1)	0.9(0.8–1.1)	0.264	-	

a Adjusted for variables that were significant at p<.2 in the bivariate analysis and data collection site;

b Pearson chi-square;

c Data not collected in Siaya DH and Tingwangi;

¥ Row percentages shown.

Of the 9,419 SARI patients who were followed up after being admitted to the hospital, 257 (2.7%) died; 246 (96%) of these deaths occurred within 30 days of admission (Table 5). The median length of stay was 4 days (IQR = 2–6 days) for all SARI patients. The percentage of SARI patients who died varied between hospitals, and SARI patients with underlying chronic disease were more likely to die than those without (Adjusted Relative Risk = 1.9, 95% CI = 1.3–2.8). There was no difference in the duration of hospitalization between influenza-positive and influenza-negative patients. However, fewer influenza-positive patients died compared to influenza-negative patients (0.9% vs. 2.8%, p = 0.010). Of the seven influenza-positive patients who died, six were male, the median age was 1 year (range: 8 months–32 years), and the median number of days between admission and death was 12 days (range 4–19 days). Three had pandemic influenza H1N1, one had H3N2, three had influenza B, and two were influenza A with indeterminate subtyping results.

**Table 5:** Relative risk of various demographic and clinical characteristics for death within 30 days of admission among SARI patients admitted to 8 hospitals in Kenya, January 2008–June 2013.

	Total (N = 9,339)	Fatal cases (N = 246)	Non-fatal cases (N = 9,093)	Unadjusted RR (95% CI)	p-value	Adjusted^a^ RR (95% CI)	p-value
		n(%)¥	n(%)¥				
**Sex**							
Male	5,171	126(2.4)	5045(97.6)	Ref			
Female	4,168	120(2.9)	4048(97.1)	1.2(0.9–1.5)	0.185	1.1(0.9–1.4)	0.379
**Site**							
Kenyatta NH	733	57(7.8)	676(92.2)	Ref		Ref	
Coast PGH	842	17(2.0)	825(98.0)	0.3(0.2–0.4)	<0.001	0.4(0.2–0.8)	0.012
Nakuru PGH	1,065	23(2.2)	1042(97.8)	0.3(0.2–0.4)	<0.001	0.5(0.3–0.9)	0.013
Nyeri PGH	1,648	25(1.5)	1623(98.5)	0.2(0.1–0.3)	<0.001	0.3(0.2–0.5)	0.000
Kakamega PGH	1,783	17(1.0)	1766(99.0)	0.1(0.1–0.2)	<0.001	0.2(0.1–0.5)	0.000
Embu PGH	153	5(3.3)	148(96.7)	0.4(0.2–1.0)	0.058	0.8(0.3–2.3)	0.726
Garissa PGH	68	6(8.8)	62(91.2)	1.1(0.5–2.5)	0.758	1.5(0.8–3.1)	0.207
Siaya DH	3,047	96(3.2)	2951(96.8)	0.4(0.3–0.6)	<0.001	0.4(0.1–1.7)	0.225
**Length of illness before presenting to hospital**							
0–3 days	4,745	97(2.0)	4648(98.0)	Ref		Ref	
4–7 days	3,901	114(2.9)	3787(97.1)	1.4(1.1–1.9)	0.009	1.3(1.0–1.7)	0.066
>7 days	693	35(5.1)	658(94.9)	2.5(1.7–3.6)	<0.001	1.6(1.1–2.3)	0.023
**Duration of hospitalization**							
0–3 days	4,466	113(2.5)	4353(97.5)	Ref		Ref	
4–7 days	3,724	82(2.2)	3642(97.8)	0.9(0.7–1.2)	0.332	0.7(0.6–1.0)	0.028
>7 days	1,149	51(4.4)	1098(95.6)	1.8(1.3–2.4)	0.001	0.9(0.6–1.2)	0.442
**Influenza test**							
Tested negative	8,585	239(2.8)	8346(97.2)	Ref			
Tested positive	754	7(0.9)	747(99.1)	0.3(0.2–0.7)	0.004	0.4(0.2–0.8)	0.010
**Underlying chronic disease**							
No underlying chronic disease	5732	114(2.0)	5618(98.0)	Ref			
Reported any underlying chronic disease	525	34(6.5)	491(93.5)	3.3(2.2–4.7)	<0.001	1.9(1.3–2.8)	0.001
**Signs and symptoms on admission** *							
Reported or documented fever (Yes vs. No)	7,954	209(2.6)	7745(97.4)	1.0(0.7–1.4)	0.925	-	
Difficulty in breathing/shortness of breath (Yes vs. No)	6,637	172(2.6)	6465(97.4)	0.9(0.7–1.2)	0.687	-	
Weight loss (Yes vs. No)	3,004	145(4.8)	2859(95.2)	3.2(2.5–4.1)	<0.001	2.1(1.6–2.9)	0.000
Convulsions (Yes vs. No)	2,413	49(2.0)	2364(98.0)	0.7(0.5–1.0)	0.032	0.8(0.6–1.1)	0.169
Stridor (Yes vs. No)^b^	1,433	49(3.4)	1384(96.6)	1.4(1.0–2.0)	0.037	1.0(0.7–1.4)	0.891
Nasal flaring (Yes vs. No)^b^	4,175	110(2.6)	4065(97.4)	1.1(0.8–1.5)	0.447	-	
Tachypnea (Yes vs. No)^b^	2,098	69(3.3)	2029(96.7)	2.0(1.4–2.8)	<0.001	1.0(0.6–1.5)	0.841
Chest in-drawing (Yes vs. No)^b^	3,552	98(2.8)	3454(97.2)	1.2(0.9–1.6)	0.159	1.5(1.1–2.0)	0.016
Grunting (Yes vs. No)^b^	2,476	80(3.2)	2396(96.8)	2.5(1.7–3.7)	<0.001	1.6(1.0–2.7)	0.070
Unable to drink or breastfeed at all (Yes vs. No)^b^	2,391	61(2.6)	2330(97.4)	1.1(0.8–1.4)	0.711	-	
Lethargic (Yes vs. No)	2,694	105(3.9)	2589(96.1)	1.8(1.4–2.4)	<0.001	1.2(0.9–1.6)	0.265
Loss of consciousness (Yes vs. No)	664	32(4.8)	632(95.2)	2.0(1.4–2.8)	<0.001	2.2(1.4–3.3)	0.000
Headache (Yes vs. No)	436	22(5.0)	414(95.0)	2.0(1.3–3.1)	0.001	1.9(1.2–3.0)	0.011
Nausea (Yes vs. No)	1,058	44(4.2)	1014(95.8)	1.7(1.2–2.4)	0.001	1.2(0.8–1.8)	0.273
Vomiting (Yes vs. No)	4,681	105(2.2)	4576(97.8)	0.7(0.6–1.0)	0.018	0.9(0.6–1.1)	0.311

a Adjusted for variables that were significant at p<.2 in the bivariate analysis and data collection site;

b Applies only for children <5 years;

¥ Row percentages shown.

*Signs such as stridor, nasal flaring, tachypnea, chest in-drawing and grunting were evaluated at the time of patient presentation to the hospital.

## Discussion

This is the first report of national influenza sentinel surveillance in Kenya. We found that throughout six years of surveillance, influenza was an important contributor to both inpatient and outpatient acute respiratory illness. Indeed, influenza was associated with nearly one-tenth of all hospitalized SARI cases, and almost one-sixth of all outpatient ILI cases. Over half of patients enrolled in our national SARI and ILI surveillance system were children less than two-years old. Although the percent-positivity for influenza was higher for both SARI and ILI in older age groups, the absolute number of influenza cases was greatest in children under age two. Similar findings were recently reported in a description of influenza surveillance in 15 African countries [10], including Kenya, from 2006–2010. Other reports from Kenya [2], [15]–[17] and studies from other resource-poor countries, like Bangladesh and Lao PDR [18], [19], have also shown that young children bear a heavy burden of influenza-associated respiratory disease.

In Kenya, influenza vaccine is rarely used [15], and in our surveillance system, although slightly more than one percent of patients had been vaccinated against influenza in the prior year, most of these patients were from Kakuma Refugee Camp and were vaccinated in 2010 as part of a free seasonal influenza vaccination campaign that was offered in the camp. Young children <2 years old could be a logical initial target population if a nationwide influenza immunization program is implemented in the future. Maternal immunization could also be considered to protect infants <6 months old [20]. Additionally, we found that nearly one-sixth of all influenza-associated SARI patients had been hospitalized in the previous 12 months, a trend that has also been documented in studies in the United States [21]. We observed this same trend among influenza-negative SARI patients. Hospitalized patients, although a much smaller population, could also be a cost-effective target for vaccination in Kenya if more influenza vaccine were to become available in the country.

Although we conducted surveillance over six years, a relatively robust time period to detect seasonal trends, we were not able to detect a consistent, discrete seasonality to influenza activity in Kenya. However, in most years, influenza activity was most pronounced during July to November, a period of low precipitation, relatively lower temperatures, and variable humidity in Kenya [22], [23], and activity was minimal during December and January, which tend to be the warmest months of the year in Kenya [22]. This same seasonal influenza pattern has been described in neighboring Uganda [24]. However, in Kenya, influenza circulated year-round and was detected during a minimum of ten months per year throughout the six year period. In some years, like 2011, influenza also circulated early in the year, during a period typical of high temperatures and heavy rains. Year-round influenza activity has been reported in other tropical countries [25]. Although the trend of increased influenza activity in the middle of the calendar year would support the use of southern hemisphere vaccine in Kenya, the relatively continuous circulation of influenza combined with the variable seasonal peaks in some years complicates this recommendation. In addition, information about the concordance between circulating strains in Kenya and southern and northern hemisphere vaccine strains, which is being evaluated in a separate analysis, will be essential to this recommendation.

In Kenya, influenza types and subtypes co-circulated through the six years of surveillance. Following the introduction of influenza A (H1N1)pdm 09 in 2009, through 2013, H3N2, A (H1N1) pdm 09, and influenza B co-circulated in Kenya, a trend that was also seen in neighboring Uganda and Rwanda [26].

We found that influenza was associated with deaths and was responsible for a considerable burden on hospitals. Of the nearly 10,000 hospitalized SARI patients we followed, 0.9% of the 754 influenza-positive SARI patients died. This case-fatality ratio for influenza-associated hospitalizations is substantial. It is higher than estimates of in-hospital case-mortality rates for children in the United States [27] and Thailand [3], although it is lower than rates recently reported for seasonal and pandemic influenza in India [28]. This may reflect a difference in underlying chronic illness or acute conditions, such as HIV or malaria, which we did not routinely test for in SARI or ILI patients. In our surveillance population, the median age was very young (16 months), and the median age of patients who died was 2 years old; this contrasts with the findings from the studies in Thailand and India, where most patients who died were adults, and many were older than 50. While there were not any reports of patients who refused to be consented, it is possible that sicker patients, particularly those who died in the hospital, were not enrolled. If sick patients came into the hospital in the evening or on weekends and died in the hospital before the next weekday morning, we would not have captured them in our surveillance system. Additionally, we would have missed patients who died shortly after arriving in the hospital, before even being admitted to a hospital ward. Therefore the CFRs we report for SARI in general and influenza-associated SARI may be under-estimates. The estimated CFR should not have been affected by the surveillance project at the hospital; surveillance officers evaluated patients on admission but were not involved in patient management.

Less than 3% of SARI patients we captured were adults; while this may reflect a lower burden of severe influenza in adults in Kenya, it likely reflects a component of relatively lower health clinic utilization in this age group [29], [30]. This disparity may also reflect the requirement of measured fever in the case definition of SARI in patients >5 years old; a recently published longitudinal cohort study in England showed that only a minority of people with PCR-confirmed influenza had fever with a temperature greater than 37.8°C[31]. Finally, the difference may reflect a possible sampling bias in our surveillance system; surveillance officers may have preferentially captured children in pediatric wards, although they were instructed to routinely survey all wards. In our surveillance system in Kenya, the median duration of hospitalization was 4 days for influenza-positive patients. This length of stay is similar to results from studies in the United States, India and Thailand [3], [27], [28].

Our findings are subject to several limitations. First, the case definition for SARI was different for children <5 years old and persons ≥5 years old, which limited our ability to compare SARI patients across age groups. In addition, we know from previous studies in Kenya that the SARI case definition does not capture all influenza cases [17], [32]; therefore we likely missed some hospitalized influenza cases. Second, we only sampled the first three ILI patients of the day. This could have biased our ILI findings towards sicker patients or patients who lived closer to the health facilities and arrived at the clinic earlier. We did not collect data regarding non-enrolled ILI patients, which would have helped us to better understand how representative sampled ILI patients were of all ILI patients. Furthermore, our surveillance system did not include specialty wards like obstetrics; therefore we were not able to collect data on the relative burden of influenza among pregnant women, a priority group for vaccination [33]. Given that pregnant women can transfer protective antibodies to infants, for whom no influenza vaccine is currently available [20], future surveillance in Kenya should monitor pregnant women. In addition, for the last 18 months, because of budgetary constraints, we scaled back our surveillance to include only nine hospitals, all but two of which conducted SARI surveillance only. Therefore, results for this period were likely not as nationally representative compared to the previous results. This change to a more limited surveillance system reflects the sustainability challenges of conducting surveillance, particularly in resource-poor settings. Finally, our surveillance system was facility-based rather than population-based. Because we were not able to determine catchment areas for each site, and because health utilization practices vary considerably in different parts of Kenya [29], [30], we were not able to determine the overall incidence of influenza in the population. We were also not able to determine the relative hospital burden of influenza because we did not collect data on overall admissions.

We found that during six years influenza contributed to hospitalized respiratory illness and outpatient ILI in all age groups year-round in 11 sites across Kenya. The absolute burden of influenza-associated respiratory illness was highest in children <2 years old; this group could be considered for prioritization for vaccine. While influenza circulated year-round in Kenya, during most years, influenza activity peaked during the peak of the southern hemisphere influenza season. Additional years of surveillance will be helpful in order to better understand the seasonality of influenza in Kenya and inform potential vaccine recommendations.
